# Data on the endogenous conversion of tyrosol into hydroxytyrosol in humans

**DOI:** 10.1016/j.dib.2019.104787

**Published:** 2019-11-12

**Authors:** Anna Boronat, Julian Mateus, Natalia Soldevila-Domenech, Mercè Guerra, Jose Rodríguez-Morató, Carlota Varon, Daniel Muñoz, Francina Barbosa, Juan Carlos Morales, Andreas Gaedigk, Klaus Langohr, Maria-Isabel Covas, Clara Pérez-Mañá, Montserrat Fitó, Rachel F. Tyndale, Rafael de la Torre

**Affiliations:** a Integrative Pharmacology and Systems Neuroscience Research Group, Neurosciences Research Program, IMIM-Institut Hospital del Mar d’Investigacions Mèdiques, Dr. Aiguader 88, 08003, Barcelona, Spain; b Department of Experimental and Health Sciences, Universitat Pompeu Fabra (CEXS-UPF), Dr. Aiguader 80, 08003, Barcelona, Spain; c Department of Pharmacy, Vall d’Hebron Barcelona Hospital Campus, Passeig de Vall d’Hebron 119-129, 08035, Barcelona, Spain; d Cardiovascular Risk and Nutrition Research Group, IMIM (Hospital del Mar Research Institute), Dr. Aiguader 88, 08003, Barcelona, Spain; e CIBER de Fisiopatología de la Obesidad y Nutrición (CIBEROBN, CB06/03/028), Monforte de Lemos 3-5, 28029, Madrid, Spain; f CAP Barceloneta, Parc Sanitari Rovira Virgili, Passeig Marítim, 25 08003, Barcelona, Spain; g Department of Biochemistry and Molecular Pharmacology, Instituto de Parasitología y Biomedicina López Neyra, CSIC, PTS Granada, Avda. del Conocimiento, 17, 18016, Armilla, Granada, Spain; h Division of Clinical Pharmacology, Toxicology, and Therapeutic Innovation, Children's Mercy Kansas City, Kansas City, MO, USA; i Department of Statistics and Operations Research, Polytechnic University of Catalonia, Barcelona, Spain; j NUPROAS Handesbolag (NUPROAS HB), Nacka, Sweden; k School of Medicine, Universitat Autònoma de Barcelona, Bellaterra, Spain; l Hospital Universitari Germans Trias i Pujol (IGTP), Badalona, Spain; m Campbell Family Mental Health Research Institute, Centre for Addiction and Mental Health, Departments of Pharmacology & Toxicology, and Psychiatry, University of Toronto, 1 King's College Circle, Toronto, ON M5S 1A8, Canada

**Keywords:** Tyrosol, Hydroxytyrosol, Endogenous conversion, *CYP2A6*, *CYP2D6*, Cardiovascular risk

## Abstract

Here we present new and original data on the endogenous conversion of tyrosol (Tyr) into hydroxytyrosol (OHTyr) in humans and its effects on the cardiovascular system. A randomized, crossover, controlled clinical trial was performed with individuals at cardiovascular risk (n = 33). They received white wine (WW) (females 1, males 2 standard drinks/day), WW plus Tyr capsules (WW + Tyr) (25mg Tyr capsule, one per WW drink), and water (control) *ad libitum*. Intervention periods were of 4 weeks preceded by three-week wash-out periods. We assessed the conversion of Tyr to OHTyr, its interaction with a polygenic activity score (PAS) from *CYP2A6* and *CYP2D6* genotypes, and the effects on cardiovascular risk markers. For further details and experimental findings please refer to the article “Cardiovascular benefits of tyrosol and its endogenous conversion into hydroxytyrosol in humans. A randomized, controlled trial” [1].

**Table:** Specifications Table

Subject	*Nutrition*
Specific subject area	*Nutritional biochemistry and genotype interaction*
Type of data	*Tables, Figures, File text*
How data were acquired	*HPLC-MS-MS for Tyr and OHTyr* *Genotyping of allelic variants of CYP2A6 and CYP2D6 .A polygenic score activity was calculated* *Reactive hyperemia index, RHI for endothelial function* *Automated methods, HPLC, and ELISA for cardiovascular risk biomarkers* *Real-time polymerase chain reaction (PCR) for gene expression*
Data format	*Raw data collection and analysis*
Parameters for data collection	*Before and after each one of the three interventions with 1) white wine (WW) (females 1, males 2 standard drinks/day), 2) WW plus Tyr capsules (WW + Tyr) (*25mg Tyr *capsule, one* per *WW drink), and 3) water (control) ad libitum. Intervention periods were of 4 weeks preceded by 3-weeks washout periods.*
Description of data collection	*Biological samples were collected and processed in the context of a randomized controlled intervention trial by field investigators*
Data source location	*Barcelona, Spain*
Data accessibility	*With the article*
Related research article	*Boronat A* et al. *Cardiovascular benefits of tyrosol and its endogenous conversion into hydroxytyrosol in humans. A randomized, controlled trial. Free Radical Biol Med, 2019 Aug 31;143:471-481.*https://doi.org/10.1016/j.freeradbiomed.2019.08.032.

**Table:** 

**Value of the Data** • The presented data provide further details on how the polygenic activity score to evaluate the efficiency of Tyr to OHTyr conversion was generated. • This report describes the effects on cardiovascular biomarkers of an intervention with white wine and white wine enriched with Tyr. • They are useful to predict the effects of nutritional interventions rich in Tyr (olive oil, wine, beer …) considering the interaction with individual's genetic background. • The present data are of interest for the effects of Tyr alone and in the future within Tyr rich foods • These data can be used for the design of new nutraceutical based on Tyr ingestion in humans

## 1 Data

**Fig. 1:**
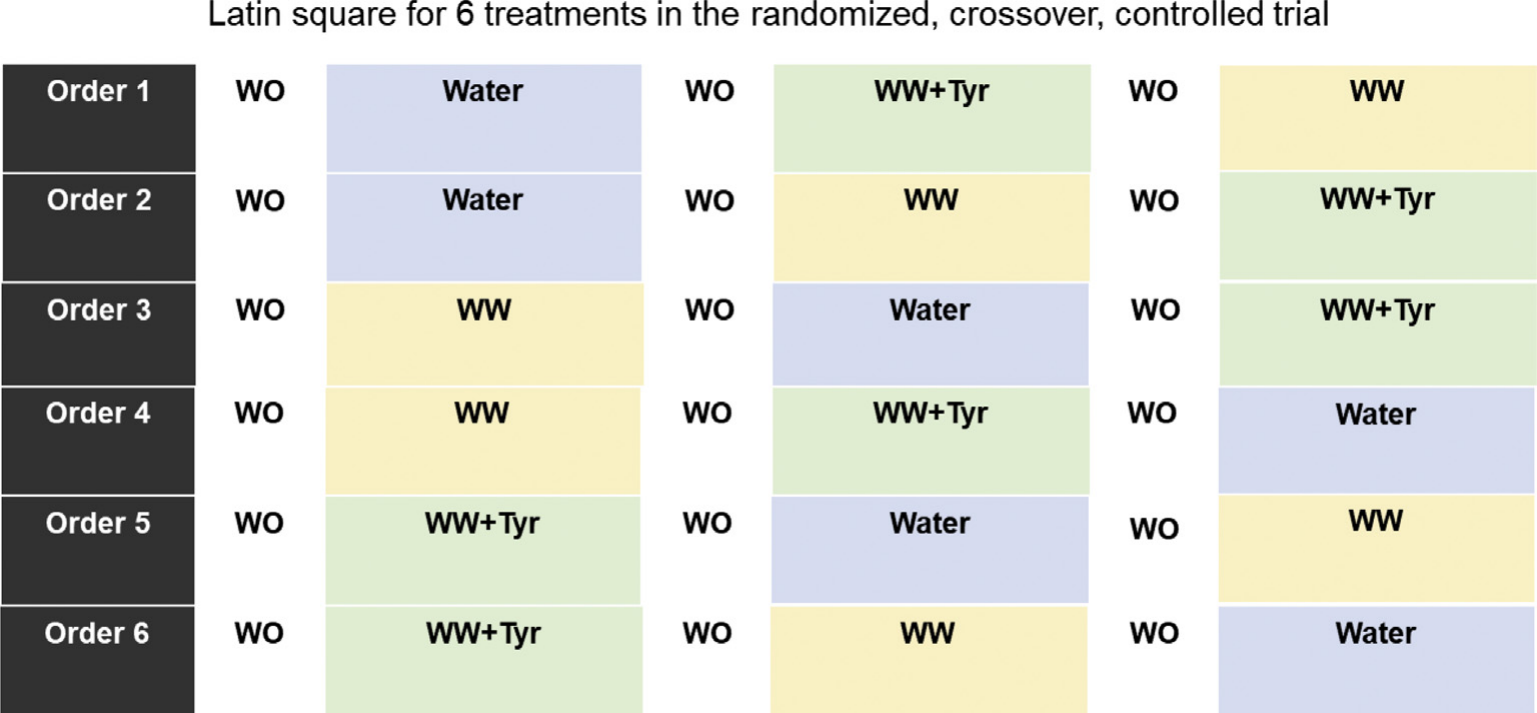
Schema of the clinical trial. Intervention periods of 4 weeks. WO: wash-out period (3 weeks) without alcohol and following a low-phenolic content diet. WW (white wine): 2 glasses (270 mL, 27 g of alcohol, 2.8 mg of Tyr and 0.4 mg of OHTyr) for men, and 1 glass (135 mL, 13.5 mg, 1.4 mg of Tyr, and 0.2 mg of OHTyr) for women. WW + Tyr (white wine plus tyrosol): 2 glasses of wine: 270 mL, 27 g of alcohol, 2.8 + 50 mg of Tyr (2 capsules), and 0.4 mg of OHTyr for men, and 1 glass:135 mL, 13.5 g of alcohol, 1.4 mg + 25 mg of Tyr (1 capsule), and 0.2 mg of OHTyr for women.

Thirty-three participants (21 men and 12 women) were randomly allocated to participate in a clinical trial, and 32 participants completed the trial. Initially, 192 subjects were assessed for eligibility, 157 were excluded for 1) not meeting the inclusion criteria, 2) refusing to participate, 3) taking medication non compatible with the interventions, 4) suffering from a coronary heart disease, 5) having undergone bariatric surgery, 6) intestinal alterations, 7) mobility problems, 8) chronic inflammatory diseases, 9) dysregulated hypertension, 10) illicit drug consumption, 11) heavy alcohol consumption, and 12) hepatic alterations. The 33 volunteers were randomly allocated to receive the following treatments for 4 weeks: WW, WW + Tyr and control intervention [1] (Fig. 1).

Baseline characteristics of the participants are shown in Table 1. No dietary differences were observed among interventions (Table 2). Volunteers were genotyped for multiple allelic variants of *CYP2A6* and *CYP2D6* (Table 3). For each enzyme, an activity score was given to each volunteer according to the alleles identified (Table 4). A pooled polygenic activity score (PAS) was calculated by adding the two activity scores together.

**Table 1:** Baseline characteristics of the participants.

Variable	Values
Age, y	65.3 ± 6.2
Gender, n (%)	
Women	12 (36.4%)
Men	21 (63.6%)
BMI, kg/m^2^	32.6 ± 4.2
LDL cholesterol, mg/dL	118 ± 34.4
HDL cholesterol, mg/dL	50.2 ± 12.9
Total cholesterol, mg/dL	192 ± 39.3
Triglycerides, mg/dL	120 ± 72.2
Cardiovascular Risk factors, n (%)	
Current smokers	6 (18.2%)
Family history of premature CHD	6 (19.4%)
Obesity (BMI ≥ 25kg/m^2^)	32 (97.0%)
Type 2 Diabetes	13 (39.4%)
Hypertension	28 (84.8%)
High LDL cholesterol (>130 mg/dL)	25 (75.6%)
Low HDL cholesterol (<40 mg/dL for men or <50 mg/dL for women)	8 (24.2%)
Medications, n (%)	
Alfa blockers	2 (6.1%)
Beta blockers	6 (18.2%)
ACE inhibitors	14 (42.4%)
Angiotensin II receptor antagonists	11 (33.3%)
Diuretics	13 (39.4%)
Statins	16 (48.5%)
Oral hypoglycemic drugs	12 (36.4%)
Acetylsalicylic acid	10 (30.3%)

Data presented as mean ± SD or n (%) (n = 33). BMI, body mass index; LDL, low density lipoproteins; HDL, high density lipoproteins; CHD, coronary heart disease.

**Table 2:** Energy, nutrients, and fiber at the beginning and at the end of the clinical trial.

Variable	Treatment						P*
	Control	P	WW	P	WW+TYR	P	
Energy, kcal/day							
Baseline	1695 ± 446		1663 ± 421		1624 ± 370		NS
12-week	1643 ± 361	0.616	1650 ± 354	0.868	1737 ± 450	0.082	
HC, % energy							
Baseline	38.2 ± 8.6		40.2 ± 6.4		38.8 ± 6.3		NS
12-week	38.3 ± 7.3	0.906	37.6 ± 7.7	0.095	37.8 ± 7.5	0.360	
HC, grams							
Baseline	159 ± 48		165± 43		157 ± 38		NS
12-week	156 ± 41	0.811	153 ± 40	0.209	163 ± 45	0.532	
Protein, % energy							
Baseline	20.9 ± 4.0		19.2 ± 3.0		21.5 ± 4.5		NS
12-week	21.2 ± 4.1	0.578	19.5 ± 4.8	0.028	19.0± 3.9	0.142	
Protein, grams							
Baseline	88 ± 25		79 ± 19		86 ± 24		NS
12-week	87 ± 25	0.939	81 ± 30	0.651	82 ± 23	0.184	
Total Fat, % energy							
Baseline	40.7 ± 7.5		40.3 ± 6.2		39.2 ± 6.6		NS
12-week	40.2 ± 7.2	0.625	36.7 ± 9.0	0.496	38.6 ± 6.3	0.230	
Total Fat, grams							
Baseline	78 ± 29		76 ± 28		72 ± 24		NS
12-week	74 ± 23	0.489	68 ± 24	0.015	76± 28	0.809	
SFA, % energy							
Baseline	11.4± 3.8		10.1 ± 3.0		11.4 ± 4.2		NS
12-week	10.7 ± 3.7	0.526	10.1 ± 3.9	0.953	10.4 ± 3.7	0.272	
SFA, grams							
Baseline	22 ± 12		19 ± 9		21 ± 12		NS
12-week	20 ± 10	0.428	19 ± 10	0.873	21 ± 11	0.855	
MUFA,% energy							
Baseline	19.8 ± 5.0		19.7 ± 4.2		18.6 ± 5.0		NS
12-week	20.1 ± 4.2	0.893	18.4 ± 5.1	0.177	19.6 ± 3.5	0.172	
MUFA, grams							
Baseline	53.3 ± 21.0		53.8 ± 15.5		53.0 ± 16.6		NS
12-week	51.9 ± 21.2	0.475	46.5 ± 13.3	0.274	50.0 ± 14.3	0.054	
PUFA, % energy							
Baseline	6.1 ± 2.6		6.8 ± 2.5		6.0 ± 2.1		NS
12-week	5.9 ± 2.3	0.697	5.2 ± 2.1	0.222	5.4 ± 1.9	0.179	
PUFA, grams							
Baseline	12 ± 6		13 ± 8		11 ± 4		NS
12-week	11 ± 7	0.901	10 ± 5	0.025	11 ± 6	0.960	
Fiber, g/day							
Baseline	20 ± 7		23 ± 11		20 ± 8		NS
12-week	20 ± 8	0.894	23 ± 11	0.848	21 ± 9	0.408	

Dietary data is expressed as mean ± SD (N = 32). HC, carbohydrates; SFA, saturated fatty acids; MUFA, monounsaturated fatty acids; PUFA, polyunsaturated fatty acids. P Intra-treatment comparisons by Student's *t*-test. *P value for ANOVA repeated measures adjusted by age and sex.

**Table 3:** Characteristics of *CYP2A6* and *CYP2D6* SNPs tested.

Tested Allelic Variants	Reference Number	Nucleotide Substitution	Amino acid substitution	TaqMan Assay ID
*CYP2A6*	rs1801272	479T > A	Leu160His	C_27861808_60
	rs28399433	- 48T > G	Upstream	C_30634332_10
*CYP2D6*	rs1135840	4181G > C	Ser486Thr	C_27102414_10
	rs16947	2851C > T	Arg296Cys	C_27102425_10
	rs3892097	1847G > A	Intron Variant	C_27102431_DO
	rs5030656	2616_2618delAAG	Lys281del	C_32407229_60
	rs1065852	100C > T	Pro34Ser	C_11484460_40
	rs769258	31G > A	Val11Met	C_27102444_80
	rs28371725	2989G > A	Intron Variant	C_34816116_20

**Table 4:** Activity score assigned to each tested variant in the PAS model.

Tested Allelic	Variants	Functional consequence	Activity score	Defining SNP
*CYP2A6*	*2	No function	0	479T > A
	*4	No expression	0	Gene deletion
	*9	Decreased	+0.5	- 48T > G
	*12	Decreased	+0.5	Hybrid allele with CYP2A7
	*1xN	Increased	+2	Multiple copies
*CYP2D6*	*2	Normal	+1	2851C > T 4181 G > C
	*4	No function	0	1847 G > A^a^
	*5	No expression	0	Gene deletion
	*9	Decreased	+0.5	2616 del AGG
	*10	Decreased	+0.5	100C > T 4181 G > C
	*35	Normal	+1	31G > A 2851C > T 4181 G > C
	*41	Decreased	+0.5	2989 G > A 2851C > T 4181 G > C
	*1xN	Increased	+2	Multiple copies
	*2xN			
	*35xN			

a *4 sub-alleles can commonly present other SNPs such as 100C > T, 4181 G > C and/or 2851C > T.

**Fig. 2:**
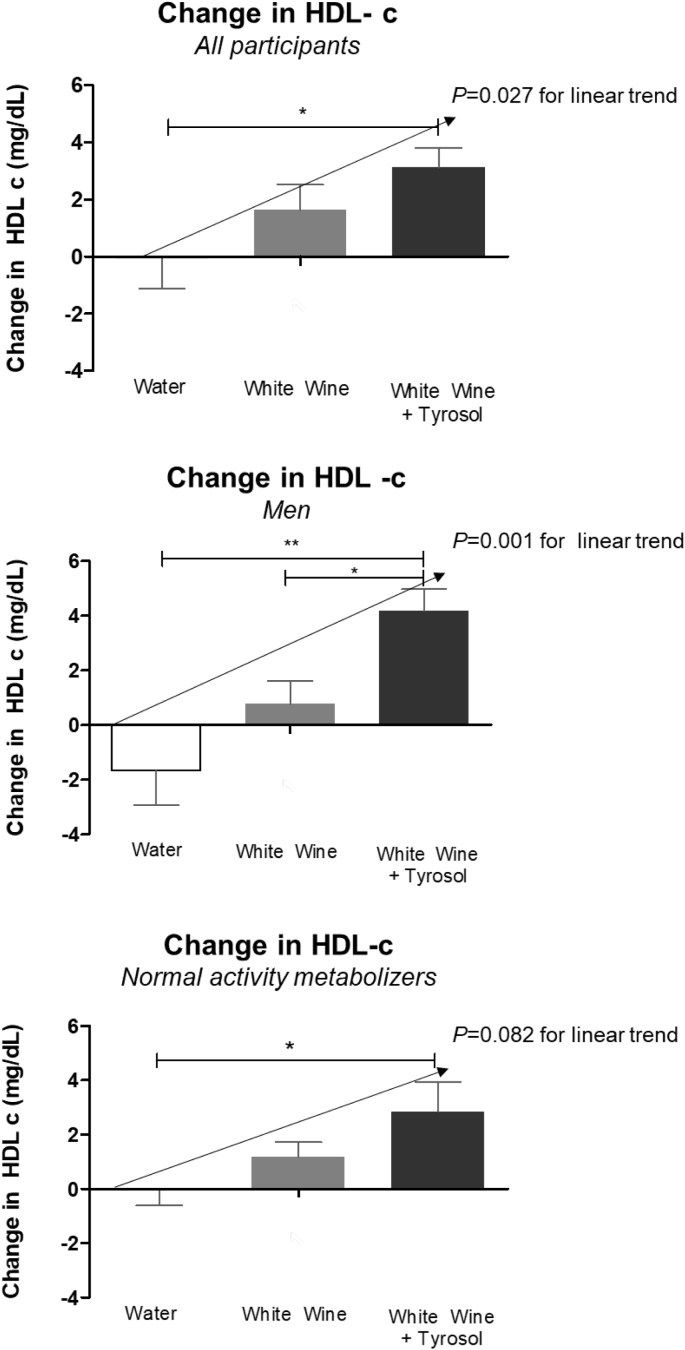
Changes in HDL cholesterol (HDL-c) after interventions. Change in HDL-c compared to the baseline of the intervention expressed as mean and SD in all participants (A), only men (B) and only normal activity metabolizers (C). ANOVA adjusted by age, sex and smoking habits, LDL cholesterol at the beginning of the clinical trial, and baseline levels * P < 0,05; **P < 0,01.

**Fig. 3:**
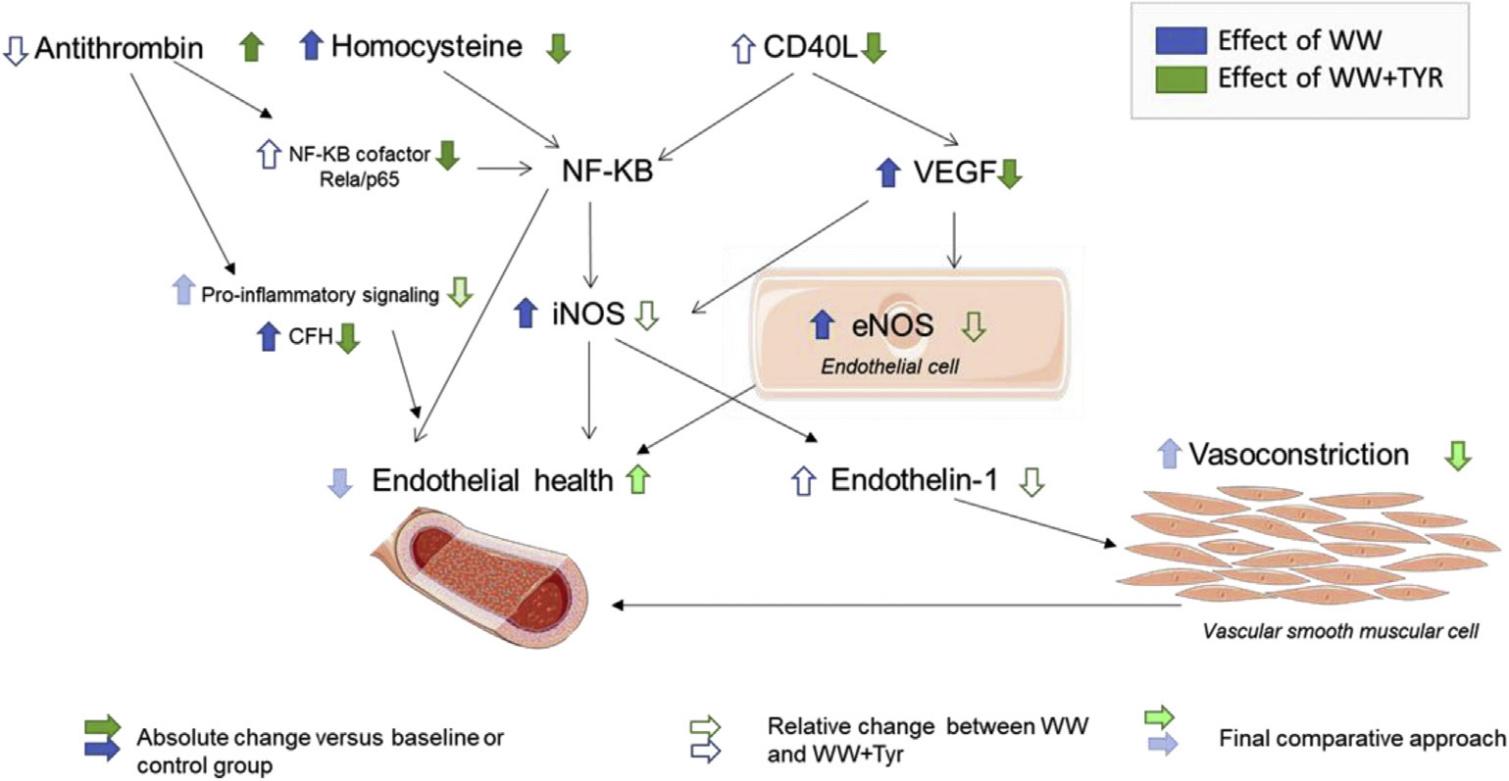
Comparison of the effects of white wine (WW) (blue) versus those of white wine plus tyrosol (WW + Tyr) (Green), CD40L, CD40 ligand; NF-KB, nuclear factor kappa B; CFH, complement factor H; iNOS, inducible nitric oxide synthase; eNOS, endothelial nitric oxide synthase.

On the basis of PAS, 11 individuals were categorized as low (LA, PAS range: 1–2.5), 19 as normal (NA, PAS range: 3–4), and 2 as rapid (RA, PAS range = 5) activity metabolizers. Due to their low number, RAs were excluded for analyses. Age and gender were equally distributed among the three groups. The conversion of Tyr into OHTyr was assessed by measuring OHTyr and Tyr urinary recovery following each treatment. No changes were observed in lipid biomarkers (Table 5) with exception of HDL-cholesterol (HDLc) which increased after WW and WW + Tyr. HDL-c increased in a dose-dependent manner with the content of alcohol plus Tyr administered in all participants (*p* = 0.027 for linear trend), among men (*p* = 0.001 for linear trend), and with a borderline significance in the NA group (*p* = 0.082) (Fig. 2). Endothelin-1 levels at the end of WW + Tyr were lower than at the end of WW intervention (Table 6). Table 7 outlines the transcriptomic changes observed in genes related with endothelial function. Changes are grouped by sex and PAS. Fig. 3 compares the effects observed in WW and WW + Tyr intervention.

**Table 5:** Changes in lipid and inflammatory biomarkers (mg/dL).

	Interventions				
	Control	WW	WW + TYR	*P* value for WW + Tyr	
				vs Control	vs WW
Total Cholesterol	1.8 ± 12.5	2.9 ± 21.8	7.7 ± 24.4	0.665	0.753
LDL cholesterol	−1.0 ± 14.1	0.1 ± 17.8	4.7 ± 22.3	0.511	0.761
Triglycerides	14.7 ± 62.0	6.0 ± 25.9	−0.9 ± 25.7	0.290	0.788
Glucose	0.9 ± 13.4	2.7 ± 10.3	2.6 ± 12.0	0.849	0.999
hsCRP	−0.2 ± 1.3	0.02 ± 0.3	−0.01 ± 0.2	0.443	0.516

Changes expressed as mean ± SD (N = 32). LDL, low density lipoprotein; hsCRP, high sensitivity C reactive protein. ANOVA adjusted by age, sex and smoking habits, LDL cholesterol at the beginning of the clinical trial, and baseline levels.

**Table 6:** Endothelin concentrations (ng/dL) after interventions.

	Interventions				
	Control	WW	WW + TYR	P value for WW + Tyr	
				vs Control	vs WW
All participants	2.15 ± 0.90	2.33 ± 1.07	2.03 ± 0.82	0.572	**0.031**
Women	2.38 ± 1.07	2.57 ± 1.11	2.12 ± 0.81	0.479	0.108
Men	2.01 ± 0.80	2.16 ± 1.08	1.99 ± 0.87	0.990	0.463
*Genotype interaction*					
LA	1.93 ± 0.69	2.25 ± 0.96	1.89 ± 0.72	0.981	0.203
NA	2.24 ± 0.95	2.48 ± 1.32	2.13 ± 1.07	0.747	0.068

Endothelin-1 concentrations are expressed as mean ± SD (N = 32). WW, white wine; WW + Tyr, white wine plus tyrosol (Tyr) capsules; LA, low activity group metabolizers; NA, normal activity group metabolizers. ANOVA adjusted by age, sex, smoking, acetylsalicylic acid consumption, and baseline levels. *P < 0.05 versus its baseline; *P* value, significance for inter-intervention comparisons.

**Table 7:** Transcriptomic changes (% change versus baseline) after interventions.

	Intervention				
	Control	WW	WW + Tyr	*P* value for WW + Tyr	
				vs Control	vs WW
*CD40L*					
All participants	8.9 ± 60.6	21.7 ± 62.3	−26.8 ± 34.2†	**0.042**	**0.003**
Women	−10.7 ± 75.4	28.8 ± 59.5	−29.9 ± 33.7*	0.743	0.063
Men	24.6 ± 47.2	20.4 ± 66.2	−25.5 ± 35.8*	**0.016**	**0.024**
*Genotype interaction*					
LA	−4.8 ± 66.7	11.1 ± 59.8	−17.1 ± 35.6	0.874	0.514
NA	20.1 ± 60.0	16.7. ± 56.5	−28.2 ± 33.9†	0.011	0.020
*P6*5/RElA					
All participants	1.1 ± 0.5	1.18 ± 0.5	0.9 ± 0.30	0.229	**0.048**
Women	−7.6 ± 54.0	26.9 ± 63.3	−16.9 ± 29.8	0.896	0.089
Men	22.9 ± 41.9	13.4 ± 48.2	−2.2 ± 28.6	0.157	0.484
*Genotype interaction*					
LA	16.7 ± 63.5	6.3 ± 45.3	−3.0 ± 43.4	0.584	0.886
NA	6.7 ± 41.1	22.5 ± 58.3	-.12,5 ± 20.9	0.414	0.054
*CFH*					
All participants	19.8 ± 57.6	28.8 ± 56.5*	−9.1 ± 51.5	0.115	**0.025**
Women	9.2 ± 57.5	48.4 ± 51.9*	11.9 ± 63.2	0.994	0.334
Men	27.9 ± 59.5	16.6 ± 58.9	−18.1 ± 40.9*	**0.013**	**0.048**
*Genotype interaction*					
LA	21.4 ± 77.7	35.2 ± 74.8	−16.7 ± 29.5	0.359	0.150
NA	22.4 ± 46.9	22.6 ± 45.1	0.0 ± 62.8	0.438	0.433
*iNOS*					
All participants	−5.0 ± 38.3	36.7 ± 82.6*	−19.7 ± 62.4	0.734	**0.007**
Women	−16.9 ± 28.8	56.1 ± 109.4	2.3 ± 24.1	0.897	0.303
Men	−2.7 ± 41.2	29.9 ± 71.3	−27.5 ± 70.4	0.470	**0.019**
*Genotype interaction*					
LA	6.3 ± 36.2	17.0 ± 66.9	−33.0 ± 52.4	0.299	0.091
NA	−4.7 ± 38.1	46.2 ± 26.7	−10.4 ± 69.6	0.996	0.080
*eNOS*					
All participants	11.7 ± 65.6	34.9 ± 72.2*	−8.2 ± 50.2	0.509	0.035
Women	13.2 ± 71.7	42.2 ± 42.1*	14.7 ± 48.8	0.997	0.565
Men	12.9 ± 65.5	26.4 ± 83.7	−20.5 ± 49.4	0.351	0.115
*Genotype interaction*					
LA	−4.8 ± 54.9	28.9 ± 77.8	−16.2 ± 62.6	0.910	0.344
NA	28.3 ± 72.9	34.8 ± 74.6	−0.2 ± 44.8	0.536	0.334
*VEGFA*					
All participants	14.3 ± 58.1	32.2 ± 69.7*	−6.2 ± 45.2	0.398	**0.045**
Women	12.6 ± 59.0	30.4 ± 68.9	−3.0 ± 61.6	0.533	0.112
Men	9.4 ± 55.4	26.2 ± 67.9	−10.0 ± 36.3*	0.870	0.500
*Genotype interaction*					
LA	26.3 ± 65.9	19.4 ± 75.6	−3.0 ± 40.6	0.497	0.665
NA	7,7 ± 57.6	27.7 ± 60.1	−1.1 ± 46.5	0.896	0.256

Changes are expressed as mean ± SD (N = 32). WW, white wine; WW + Tyr, white wine plus tyrosol (Tyr) capsules; LA, low activity group metabolizers; NA, normal activity group metabolizers. *CD40L,* CD40 ligand; *CFH,* complement factor H; e*NOS,* endothelial nitric oxide synthase 3; i*NOS*, inducible nitric oxide synthase; *p6*5/RELA, transcription factor p65 (RELA); *VEGFA,* vascular endothelial growth factor. ANOVA adjusted by age and sex. *P < 0.05, ^†^*P* < 0.001 versus its baseline; *P* value, significance for inter-intervention comparisons.

## 2 Experimental design, materials, and methods

### 2.1 Study design

A randomized, controlled, clinical trial with 33 individuals at cardiovascular risk (21 men and 12 women) was performed (Fig. 1). Inclusion criteria were to be at high risk for coronary heart disease (CHD) with 3 or more risk factors including: current smoking (>1 cig/day during the last month), hypertension (≥140/90 mmHg or antihypertensive medication), high LDL cholesterol (>130 mg/dl or lipid-lowering therapy), low HDL-cholesterol (≤40 mg/dl in men and ≤50 mg/dl in women), overweight/obesity (body mass index ≥25 kg/m^2^), a family history of premature CHD, and/or type II diabetes treated with oral hypoglycemic agents; and to have a social or recreational use of ethanol/wine consumption at least once during lifetime. Exclusion criteria were participants with a history of cardiovascular disease or severe chronic illness, chronic inflammatory diseases, BMI > 40 kg/m^2^, suffered from any severe illness or undergone major surgery in the last three months prior to the clinical trial, an alcohol consumption exceeding 8 units or 80 g per day, a history of alcohol hypersensitivity/intolerance, illicit drug consumption, intake of antioxidant supplement(s), the taking of sedative drugs that could potentially interact with alcohol, multiple allergies or intestinal diseases, being vegetarian of following special diets, a history of food allergies or intolerances, illiteracy, and any condition that limited mobility making trial visits impossible or worsening adherence to treatments.

Participants were asked to follow a controlled diet with a moderate content of antioxidants and to abstain of any alcoholic drinks (except in the framework of treatment allocations) thorough the trial. The consumption of certain food was limited to a maximum of 1) vegetables (including pulses): one serving/day, 2) fruits (or juices): 2 pieces/day, 3) ordinary olive oil: maximum 25 mL/day, 4) drinks containing xanthines (coffee, tea, cola, energy drinks …): maximum 3 cups/day, 5) chocolate: maximum one piece (small 15 gr)/day, 6) nuts: maximum 30 g (a small handful)/week, and 7) fish: maximum 3 times per week (150 g/serving).

During the control intervention participants were allowed to only drink water (no alcohol. wine or supplemented Tyr or OHTyr). The reason for different WW doses for men and women was in order to follow the American Heart Association (AHA) guidelines, which limit alcohol consumption to one drink in women and two in men, preferably taken at meals [2]. The doses of wine administered are within those recommended by the AHA.

A 24 h food recall was performed to assess dietary intake before and after each intervention period. Physical activity was recorded at the beginning and end of the clinical trial and assessed by the Minnesota Leisure Time Physical Activity Questionnaire, validated for the Spanish population [3]. A general physical examination, and routine urine, blood chemical and hematological analyses, were performed at the beginning and end of the trial. Blood and 24h-urine samples were collected at fasting state before and after each intervention period. Blood was collected into 10 mL tubes containing EDTA and centrifuged (1700g, 15 min, 4 °C), and plasma and buffy coat samples were then isolated. Peripheral blood mononuclear cells (PBMC) were isolated using a Vacutainer Cell Preparation Tube (CPT™) and kept for RNA extraction. Plasma, urine, and PBMC samples were frozen at −80 °C until analysis. Genomic DNA isolation from buffy coat was performed with QIAamp DNA Blood Midi Kit (Qiagen, Dusseldorf, Germany).

### 2.2 Tyr and OHTyr metabolites analysis

The urinary concentrations of Tyr and OHTyr metabolites were determined from samples collected before and after each intervention following a validated methodology [4]. Briefly, 0.5 mL of urine was diluted with 0.5 mL of purified water, spiked with 10 μL of internal standard mixture (containing 10 μg/mL of 3-(4-hydroxyphenyl)-1-propanol, 3-(4-hydroxyphenyl)-1-propanol glucuronide and 10 μg/mL HT-1′-*O*-sulfate) and stabilized with 1 mL of phosphoric acid 4%. Thereafter, samples went under a solid-phase extraction using Oasis HLB columns 3 mL, 60-mg cartridges from Waters Corporation (Milford, MA USA). First, samples were loaded into cartridges then washed with 2 mL of purified water. Thereafter, the compounds of interest were eluted from the cartridge with 2 mL of pure methanol. The methanol extracts were then evaporated until dryness under a stream of nitrogen (29 °C, 10–15 psi). Finally, the dried extracts were reconstituted with a mixture of mobile phases (95% A and 5% B v/v), transferred into HPLC microvials, and analyzed using LC-MS/MS. To prepare blank samples and calibration curves, urine from volunteers after consuming a diet poor in Tyr and OHTyr- Tyr and OHTyr metabolite concentrations were below quantification limits in these blank samples. Blank urine was spiked with increasing concentrations of the metabolites of interest, and then processed in the same manner as samples (described above). Identification and quantification of the metabolites was performed using an Agilent 1200 series HPLC system coupled to a triple quadrupole (6410 Triple Quad LC/MS) mass spectrometer with an electrospray interface from Agilent Technologies (Santa Clara, CA, USA). For the chromatographic separation, an Acquity UPLC®BEH C18 column (100 mm × 3.0 mm i.d., 1.7 μm particle size) from Waters Corporation (Milford, MA, USA) was used at 40 °C. The composition of mobile phase A was 0.01% (v/v) formic acid in water, and mobile phase B was acetonitrile with 0.01% (v/v) of formic acid. Injection volume was 10 μL and the flow rate was set at 0.25 mL/min. The ion source operated in negative ionization for 27 minutes. Finally, urinary concentrations of each metabolite were standardized with the total urinary excretion volume to obtain the total recovery of each metabolite. Quantified Tyr metabolites included Tyr -4-sulfate and Tyr -4-glucuronide. OHTyr metabolites quantified were OHTyr -3-sulfate, OHTyr-4-sulfate, OHTyr-acetate-3-sulfate, OHTyr-3-glucuronide, OHTyr-4-glucuronide, and homovanillyl alcohol (HVAL)-4-glucuronide. Total Tyr and total OHTyr correspond to the molar sum of their respective quantified metabolites.

### 2.3 Genotyping

Volunteers were genotyped for multiple allelic variants of *CYP2A6* and *CYP2D6* using TaqMan genotyping assays (Applied Biosystems, Foster City, CA, USA) SNP genotyping was performed with a TaqMan allelic discrimination system (Applied Biosystems, Foster City, CA, USA). Copy-number variations (*CYP2A6* *4, *12, and CYP2D6 *5, and duplications) were analyzed with specific copy number assays. When these allelic variants were not detected, a designation of *1 (e.g. wildtype) was assigned.

#### 2.3.1 SNP genotyping

Table 3 shows the characteristics of *CYP2A6* and *CYP2D6* tested allelic variants. The following SNPs were analyzed: for *CYP2A6* *2 and *9, and for *CYP2D6* *2, *4, *9, *10, *35, and *41. TaqMan SNP genotyping assay were used, which included FAM™ and VIC™ dye-labeled TaqMan pre-designed proves specifics for each SNP. PCR was performed in a QuantStudio™ 12K Flex Real-Time PCR System (Applied Biosystems, Foster City, CA, USA). Reactions were prepared with 10 ng of DNA, 0.25 μL of TaqMan SNP Genotyping Assay, and 2.5 μL of TaqMan Genotyping Master Mix (Applied Biosystems, Foster City, CA, USA). SNP determination was made using allelic discrimination plots with TaqMan Genotyper Software (Applied Biosystems, Foster City, CA, USA).

### 2.4 Copy number variation (CNV) detection analysis

TaqMan CNV assays were used to analyze *CYP2A6* allelic variants *4, *12 (Hs07545274_cn; Hs07545275_cn), and *CYP2D6* allelic variants *5 (deletion),*1xN, *2xN, and *35xN (Hs00010001_cn). Real time qPCR was performed using the specific TaqMan assays. Quantitative PCR was performed in QuantStudio™ 12K Flex Real-Time PCR System (Applied Biosystems, Foster City, USA). Reaction was carried in 384-well plates with a mixture of TaqMan Master Mix (Applied Biosystems, Foster City, CA, USA), CNV assays, 10 ng DNA/well and RNase P as reference (Applied Biosystems, Foster City, CA, USA). Reactions were performed in duplicates. Copy number calls were made with the Expression Suite Software v1.0.3 (Applied Biosystems, Foster City, CA USA). *CYP2D6* gene duplications as previously described [5]. First, a specific 6.6 kb long piece of *CYP2D6* was amplified. Second, a 3.5 kb fragment was amplified from alleles carrying gene duplications. Every duplication-positive sample was further analyzed using two long-range PCR reactions that allow to discriminate among CYP2D6*1xN, *2xN, and *4xN duplications to determine allele-defining SNPs.

### 2.5 Polygenic activity score

Tested allelic variants were categorized into: those with no-function (CYP2A6 *2, *4; CYP2D6 *4,*5); decreased function (CYP2A6 *9,*12; CYP2D6 *9,*10,*11); normal function (CYP2A6 *1; CYP2D6 *1,*2,*35); and increased function (CYP2A6 *1xN; CYP2D6 *1xN,*2xN,*35xN). A score of 0, 0.5, 1 or 2 was assigned for the presence of each allele (Table 4). For each enzyme, an activity score was given to each volunteer according to the identified alleles and classified as detailed above based on the method described by Gaedigk et al. [6]. A pooled polygenic activity score (PAS) was calculated by adding together the activity scores of both enzymes. Finally, according to their PAS, individuals were placed into three groups of predicted activity: low (LA), normal (NA), and rapid activity (RA) groups.

### 2.6 Endothelial function measurement

Endothelial function was assessed before and after interventions by monitoring endothelium-mediated changes (reactive hyperemia index, RHI) in the digital pulse waveform, known as the Peripheral Arterial Tone (PAT) signal (EndoPAT 2000; Itamar Medical Inc., Caesarea, Israel). Specially designed finger probes were placed on the middle finger of each subject's dominant hand. The probes comprised a system of inflatable latex air cuffs connected by pneumatic tubes to an inflating device controlled through a computer algorithm. A constant counter pressure (pre-determined by baseline DBP) was applied through the air cushions. Pulsatile volume changes of the distal digit induced pressure alterations in the finger cuff, which were sensed by pressure transducers and transmitted to and recorded by the EndoPAT 2000 device. EndoPAT 2000 also provides the augmentation index (AI), a measurement of arterial stiffness via pulse-wave analysis, which was normalized to 75 bpm heart rate. Measurements were performed by a trained professional with the participants in resting supine conditions, in a quiet room at a constant temperature after 10 minutes of stabilization. Hyperemic reactivity index measured by EndoPAT 2000 has been shown to predict cardiovascular disease [7].

### 2.7 Gene expression measurements

On the basis of their relationship with endothelial health and atherosclerosis, and the available data of gene expression response after VOO ingestion several candidate genes were selected. Candidate genes were AKT serine/threonine kinase 2 (*AKT2*), arachidonate 5-lipoxygenase (*ALOX5*), CD40 ligand (*CD40L*), complement factor H (*CFH*), endothelial nitric oxide synthase 3 (e*NOS*), endothelial plasminogen activator inhibitor (*SERPINE1*), inducible nitric oxide synthase (i*NOS*), interferon gamma (*IFNG*), interleukins (IL)1B (*IL1B*) and 6 (*IL6*), matrix metalloproteinases (*MMP*) 2 (*MMP2*) and 9 (*MMP9*), mitogen-activated protein kinase 14 (*MAPK14*), monocyte chemoattractant protein 1 (*MCP1*), nuclear factor (NF) (erythroid-derived 2)-like 2 (*NEF2L2*), NF-kappa B inhibitor alpha (*NFKBIA*), platelet-derived growth factor subunit B (*PDGFB*), peroxisome proliferator-activated receptor alpha (*PPARɑ*), sirtuins *(SIRT)* 1 (*SIRT1*), 2 (*SIRT2*), and 6 (*SIRT6*), transcription factor p65 (p65/RELA), tumor necrosis factor alpha (*TNF*-ɑ), and vascular endothelial growth factor (*VEGFA*). Glyceraldehyde 3-phosphate dehydrogenase (GAPDH) and B-actin were used as endogenous controls to correct changes in gene expression. Isolation of RNA from PBMC was performed with the RNeasy Mini Kit (Qiagen, Duesseldorf, Germany). DNA complementary conversion was then carried out with the High-Capacity cDNA Reverse Transcription Kit (Applied Biosystems, Foster City, CA, USA).

Gene expression was measured, before and after interventions, by a real-time polymerase chain reaction with a QuantStudio™ 12K Flex Real-Time PCR System (Applied Biosystems, Foster City, CA, USA) and SYBR Green dye-based analysis. Samples were analyzed in duplicate. Results were obtained with the Expression Suite Software v1.0.3 (Applied Biosystems, Foster City, CA, USA). Changes in gene expression were assessed first by calculating the relative quantification, applying the 2^–ΔΔCT^ of each sample. Thereafter, the fold change of each intervention was extracted by calculating the ratio between values at the end and at baseline of each intervention period.

### 2.8 Sample size and power analyses

A total sample of 32 participants would allow at least 80% power to detect a statistically significant difference among groups of 0.205 units in the RHI measurement, assuming a dropout rate of 5% and type I error of 0.005 (2-sided). The standard deviation of the measurement was assumed 0.4 [7].

### 2.9 Statistical analyses

Normality of continuous variables was assessed by normal probability plots and data were log transformed when required. Intra-treatment comparisons were assessed by Student's *t*-test for paired samples. Comparisons among treatments were made by an ANOVA for repeated measures and adjusted by age, gender, smoking, AAS medication, and baseline concentrations. In the case of lipids an additional adjustment for LDL cholesterol values at the beginning of the clinical trial was performed. A general lineal model was used to assess linear and quadratic trends. For the post-hoc pairwise comparison, the Tuckey test was used. Statistical analyses were performed with R (R Foundation for Statistical Computing, Vienna, Austria). version 3.0.2., and R package multcomp. Significance was defined as *p* < 0.05.
